# Development of Prediction Model and Experimental Validation in Predicting the Curcumin Content of Turmeric (*Curcuma longa* L.)

**DOI:** 10.3389/fpls.2016.01507

**Published:** 2016-10-06

**Authors:** Abdul Akbar, Ananya Kuanar, Raj K. Joshi, I. S. Sandeep, Sujata Mohanty, Pradeep K. Naik, Antaryami Mishra, Sanghamitra Nayak

**Affiliations:** ^1^Center of Biotechnology, Siksha O Anusandhan UniversityBhubaneswar, India; ^2^School of Life Sciences, Sambalpur UniversitySambalpur, India; ^3^Department of Soil Science, Orissa University of Agriculture and TechnologyBhubaneswar, India

**Keywords:** turmeric, artificial neural network, prediction, optimization, curcumin content

## Abstract

The drug yielding potential of turmeric (*Curcuma longa* L.) is largely due to the presence of phyto-constituent ‘curcumin.’ Curcumin has been found to possess a myriad of therapeutic activities ranging from anti-inflammatory to neuroprotective. Lack of requisite high curcumin containing genotypes and variation in the curcumin content of turmeric at different agro climatic regions are the major stumbling blocks in commercial production of turmeric. Curcumin content of turmeric is greatly influenced by environmental factors. Hence, a prediction model based on artificial neural network (ANN) was developed to map genome environment interaction basing on curcumin content, soli and climatic factors from different agroclimatic regions for prediction of maximum curcumin content at various sites to facilitate the selection of suitable region for commercial cultivation of turmeric. The ANN model was developed and tested using a data set of 119 generated by collecting samples from 8 different agroclimatic regions of Odisha. The curcumin content from these samples was measured that varied from 7.2% to 0.4%. The ANN model was trained with 11 parameters of soil and climatic factors as input and curcumin content as output. The results showed that feed-forward ANN model with 8 nodes (MLFN-8) was the most suitable one with *R*^2^ value of 0.91. Sensitivity analysis revealed that minimum relative humidity, altitude, soil nitrogen content and soil pH had greater effect on curcumin content. This ANN model has shown proven efficiency for predicting and optimizing the curcumin content at a specific site.

## Introduction

Turmeric (*Curcuma longa* L.) is a unique plant combining properties of a spice, colorant, cosmetic and a drug useful in a number of diseases. It is used as spice, herbal medicines, dyeing agents and cosmetics since vedic age (Salvi et al., 2000; Shirgurkar et al., 2001). The significance of turmeric in health and nutrition has greatly been recognized since the discovery of the pharmaceutical properties of naturally occurring phenolic compounds in it. It has been found that the dried rhizome of turmeric is a rich source of beneficial phenolic compounds known as the curcuminoids (Srinivasan, 1953; Lechtenberg et al., 2004). Curcuminoids are the most important components of turmeric, which refer to a group of phenolic compounds, chemically related to its principal ingredient, curcumin. Extensive investigation over the last five decades has indicated that curcumin reduces blood cholesterol (Rao et al., 1970; Patil and Srinivasan, 1971), prevents LDL oxidation (Ramirez-Tortosa et al., 1999), inhibits platelet aggregation (Srivastava et al., 1986, 1995), suppresses thrombosis (Srivastava et al., 1985) and myocardial infarction (MI) (Dikshit et al., 1995), suppresses symptoms associated with type II diabetes (Srinivasan, 1972), rheumatoid arthritis (Deodhar et al., 1980), multiple sclerosis (Natarajan and Bright, 2002) and Alzheimer’s disease (Lim et al., 2001).

Lack of requisite high curcumin containing genotypes and variation in the curcumin content of turmeric at different agro climatic regions are the major stumbling blocks in commercial production of turmeric. Identification of genetically superior turmeric with high curcumin content would not be possible by simple chemotyping as curcumin production is largely influenced by environmental factors. A wide range of variation in the curcumin content of turmeric at different agroclimatic regions has been reported by Singh et al. (2013). Even a same turmeric variety (cv. Suroma) has shown variation in curcumin content when grown at different agroclimatic regions of Odisha, India (Sandeep et al., 2016). Hence, it would be necessary to analyze soil nutrients and climatic factors of different agro climatic regions of Odisha with respect to high curcumin content. For optimizing environmental parameters, artificial neural network (ANN) seems to be a good model to map genome environment interaction basing on the curcumin content, soil and climatic factors to predict the proper regions/site for optimum curcumin content.

The use of ANNs has gained increasing applications where the dependency between dependent and independent variables is either unknown or very complex (Almeida, 2002; Altun et al., 2007; Khazaei et al., 2008). Neural network models provide accurate results for complicated system analysis than conventional mathematical models (Alam and Naik, 2009). Some ANN applications to solve agricultural problems include the prediction of seeding dates (Major et al., 1996), crop yield (Cerrato and Blackmer, 1990; Drummond et al., 2003; Heinzow and Tol, 2003) etc. The ANN based prediction model has also been developed for prediction of plant metabolite like podophyllotoxin content in *Podophyllum hexandrum* (Alam and Naik, 2009) and artemisinin content in *Artemisia annua* (Pilkington et al., 2014).

This study shows that the developed ANN model is a suitable way of predicting curcumin content at a particular site and to optimize the curcumin content at a specific site by modifying the changeable factors.

## Materials and Methods

### Plant Materials and Sample Stations

Turmeric samples were collected from 119 sites covering 8 agroclimatic regions at different altitudes (2.8 m -872 m) from different districts of Odisha, from May to July, 2013. From each site, representative plant samples were collected in replicates of three. The interval between two consecutive replicates was 2 m -5m. The collected fresh rhizomes were washed with running tap water to remove the soil particles, followed by washing with double distilled water. The washed rhizomes were then air dried and used for curcumin estimation. Soil samples from each sampling site were collected in replicates and brought to the laboratory for analysis of soil nutrients. From each sampling site, the data on environmental factors such as temperature, humidity and rainfall were taken as monthly averages from May, 2012 to February, 2013 and properly documented.

### Analysis of Curcumin Content

Rhizomes of turmeric were collected from field before onset of dormancy (i.e., November) for extraction of curcumin. The rhizomes were cleaned thoroughly with water, cut into small pieces and air dried. The air dried rhizomes were powdered in mortar with a pestle, 0.1 g of powdered rhizome was taken in a flat bottom flask and 75 ml of acetone was added and refluxed for 4 h. The refluxed residue was cooled, filtered and washed with 100 ml of acetone. The absorbance of the diluted sample and that of the standard curcumin (95% HPLC Purified, Charak) solution were measured at 420 nm by spectrophotometer (Thermo Scientific, Evolution 220 UV Visible) and curcumin percentage of the sample was estimated according to the ASTA method (American Spice Trade Association, 1997).

$$\text{Curcumin(\%)}=\frac{(absorbance\,\;of\,\;the\,\;sample)\times dilution\,\;factor}{factor\,\;derived\,\;from\,\;s\,\tan dard\times weight\,\;of\,\;the\,\;sample}$$

### Quantitative Analysis of Soil

The soil samples were collected in replicates of three from each site. Soil samples were collected from interior inter-row area of turmeric growing region from 0 to 15 cm depth of each agroclimatic zone. Soil samples were thoroughly mixed and stored in zip-lock polythene bags to maintain the moisture level similar to the field conditions. Samples were then stored in the laboratory for 2–3 weeks. About 200 g of soil were collected and then sieved through a 2 mm mesh. The fine soil was used for nutrient analysis.

pH of the soil samples was determined in 1:2 soil:water suspension after equilibration for half an hour with intermittent stirring using the Systronics pH meter (Model MKVI).

Total nitrogen was determined by using alkaline KMnO_4_ method (Subbiah and Asija, 1956). Hundred milliliter of 0.32% KMnO_4_ solution was added to 20 g of soil sample in an 800 ml Kjeldahl flask and then 2.5% NaOH solution was added with some distilled water. Distillation was continued and was collected at receiver tube in the 250 ml conical flask containing 20 ml boric acid (2%) with mixed indicator. The distillate was titrated against 0.02N H_2_SO_4_ taken in burette to a pink color end point and available nitrogen was calculated.

Total phosphorus in the surface soil samples were determined using Brays No-1 method. 2 g soil was extracted with 40 ml of Bray’s-1 solution (0.03NH_4_F and 0.025N HCl) and was shaken for 5 min by mechanical shaker and filtered through Whatman filter paper. 0.5 ml aliquot was transferred into a 25 ml flask 0.5 ml of ammonium molybdate solution was added and distilled water was added to make the volume up to 25 ml. Diluted SnCl_2_ (0.5 ml was diluted to 66 ml) was added and the volume was made up to the mark. Phosphorous concentration was analyzed by the help of spectrophotometer (Model: Systronics 166) at 660 nm. The concentration was calculated from the standard graph prepared by taking different phosphorous concentration. Available phosphorous in the profile soil samples were determined by extracting the soil with Olsen’s reagent (0.5 M NaHCO_3_, pH 8.5). The phosporous concentration was determined colorimetrically following the procedure of ascorbic acid reduced sulphomolybdic acid blue color in sulphuric acid system at 882 nm by using spectrophotometer (Model: Systronics 166) (Olsen and Sommers, 1982). Soil potassium content was determined by taking 5 g of soil sample in 100 ml conical flask and 25 ml of 1N NH_4_OAc solution was added to it. Then it was shaken with the help of a mechanical shaker for 5 min and the potassium concentration in the filtrate was analyzed with the help of a flame photometer (Model:Systronics 128).

Organic carbon (OC) content of the soil was determined by Wet digestion procedure of Walkley and Black as outlined in soil chemical analysis (Jackson, 1973).

### Statistical Analysis

The relationship between the curcumin content with the individual parameter of soil and climatic factors was investigated using simple linear regression model. Minitab statistical package was used for the regression analysis. The degree and nature of relationship was estimated applying pearson correlation coefficient (*r*-value). Further the statistical significance of the relationship was measured at different probability level such as *p* = 0.05, *p* = 0.01 and *p* = 0.001.

### Artificial Neural Network Model Development

For predictions and classifications of data neural networks are the useful tools. The main structure of the ANN is made up of the input layer and the output layer (Yang et al., 2014). For a complicated system a neural network model can determine the input–output relationship based on the strength of their interconnections presented in a set of sample data (Mark et al., 2000). Data approximation and signal filtering functions can be provided by such a model (Clifford and Lau, 1992). In this case, a back propagation (BP) neural-network model was created using STATISTICA software (statsoft) and trained using the environmental parameters and soil parameters as the inputs and curcumin content as the output.

**FIGURE 1:**
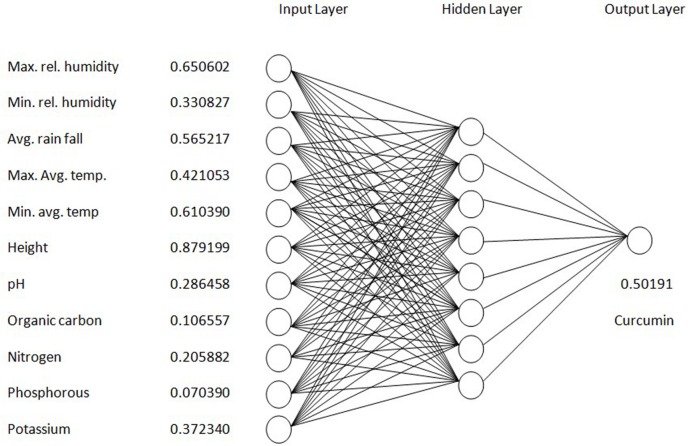
**Layers and connections of a feed-forward back-propagating ANN model for curcumin yield**.

The structure of the neural network model consisted of 11 input neurons in the input layer and one output neuron in the output layer to match the 11:1 input – output pattern of the training data set. For the neural network determined by a trial and error method, one hidden layer with eight neurons was the best topology (**Figure 1**). The neural- network model was trained in an iterative training process using the obtained training set as follows:

i = {0.650602 0.330827 0.565217 0.421053 0.610390 0.879199 0.286458 0.106557 0.205882 0.070390 0.372340 0.50191}
The first five values are the climatic factors. The sixth one refers to altitude. The next five values are the soil parameters and the last one is the curcumin content of rhizomes collected from the corresponding sites. The order of input–output data pair was randomized before the training process to avoid possible bias. In the process of training, the BP training differentiate the estimated output value with the target value connecting all the neurons to reduce the difference between the estimated and target values until the error is smaller than a predefined level. The established model was trained with the input data for an epoch of 12,000 with 0.1 learning rate. After completing the training process, the interconnection strengths between neighboring neurons are fixed and the neural network model will be capable of mapping input variables to an estimated output promptly and accurately.

The input variables in this model were normalized based on their possible ranges to avoid data saturation using the following equation.

$$a_{norm}=\left(a-a_{\min}\right)/\left(a_{\max}-a_{\min}\right)$$

Where a, a_min_, a_max_ and a_norm_ are the real valued input variable, the minimum and maximum values of the input variable and its normalized value respectively. The output from this model is an indexed value that corresponds to the input variable. To get the real-valued output, the indexed output value needs to be denormalized according to the following equation:

$$b=b_{norm}*\left(b_{\max}-b_{\min}\right)+b_{\min}$$

Where b, b_min_, b_max_ and b_norm_ are the real-valued output variable, the minimum and maximum possible values of the real-valued output and the indexed output value from the ANN model respectively.

## Results and Discussion

### Development of Artificial Neural Networks Model

For predictions and optimization neural networks are useful tools. In this case, a back-propagation (BP) neural-network model was created using STATSTICA software and trained using the environmental and soil parameters as the inputs and curcumin content as the output. The structure of the neural network model consisted of 11 input neurons in the input layer and one output neuron. One hidden layer with eight neurons was the optimal topology for the neural network determined by a trial and error method (**Figure 1**). Heinzow and Tol (2003) and Khazaei et al. (2008) have chosen four-layer backpropagation networks with two hidden layers for prediction of crop yield. These results again confirm that given sufficient hidden layer and neurons multi-layer feed-forward network architectures can approximate virtually any function of interest to any desired degree of accuracy. Mohammadi et al. (2010) devised ANN models to estimate yield level of kiwi fruit production in Mazandaran province of Iran. They used annual energy consumption per hectare of fruit production by different inputs as input variables and the yield level of fruit as output parameter. From this study they concluded that the ANN model with 6-4-1 structure was the best model for predicting the kiwi fruit yield in the surveyed region. Rahman and Bala (2010) reported that a model with 6-9-5-1, i.e., a network having an input layer with six neurons, two hidden layers with 9 and 5 neurons and one neuron in the output layer, was the best model for predicting jute production in Bangladesh. Pahlavan et al. (2012) developed various ANN models to estimate the production yield of greenhouse basil in Iran.

**FIGURE 2:**
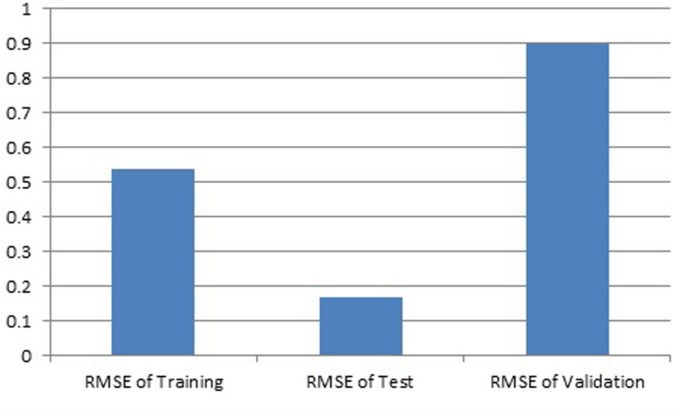
**Training, test and validation RMSE for developed ANN model**.

Results showed that feed-forward neural networks trained by BP algorithm had a good ability for creating of non-linear mapping between input and output parameters. Among the various structures model of good performance was produced by the MLFN-8 (**Figure 1**). Before arriving at this optimum, several tests were carried out with different configurations of the neural network. The configuration that had a minimal dimension and which gave satisfying results was retained with trial and error method. The number of hidden layers and neurons had a large effect on model performance. It was found that increasing the number of hidden layers increased the modeling capability. For the final network, 11-8-1 structure, the training, testing and validation RMSE values were 0.54, 0.17 and 0.9 respectively (**Figure 2**). Ideally, the RMSE values should be close to zero, indicating that on an average there were appropriate training and prediction performances.

**FIGURE 3:**
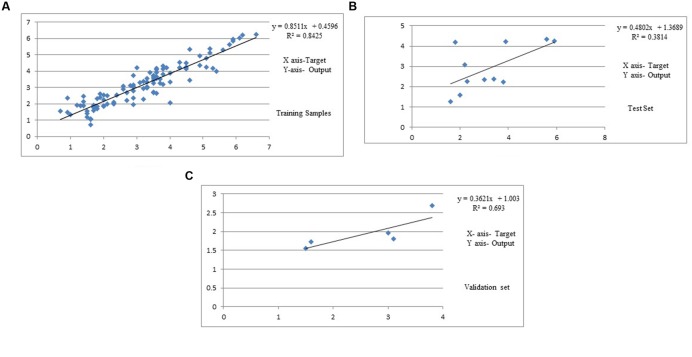
**(A)** Predicted and experimental curcumin yield of training set data. **(B)** Predicted and experimental curcumin yield of test set data. **(C)** Predicted and experimental curcumin yield of validation set data.

Training results of MLFN-8: Training results of MLFN-8 model were obtained from the experiments. In training process, the comparison result between predicted values and actual values is presented in **Table 1**. A plot between target (actual) values and output (predicted) values is provided in **Figure 3A**. The relationship between predicted values and actual values implies that the training process is precise.

**Table 1:** Predicted and experimental curcumin yield of training set data.

District	Accessionno.	Experimentalcurcumin yield(x_1_)	Predictedcurcumin yield(x_2_)	Absolute =Ix_1_-x_2_I
Kandhamal	Cr1	3.6	3.7	0.06
	Cr3	1.9	2.6	0.71
	Cr4	1.8	1.7	0.1
	Cr5	1.9	2.4	0.51
	Cr6	2.1	2	0.09
	Cr7	1.6	1.1	0.54
	Cr8	0.4	0.9	0.56
	Cr9	1.3	1.9	0.59
	Cr10	2.6	2.9	0.29
Gajapati	Cr12	1	1.3	0.35
	Cr13	3.5	3.6	0.09
	Cr14	3.6	3.9	0.33
	Cr16	1.7	1.6	0.12
	Cr17	3.3	3	0.33
	Cr19	2.4	2.5	0.15
	Cr20	2.3	2	0.31
	Cr21	3.5	3.3	0.23
	Cr22	4.5	4.1	0.37
	Cr23	3.8	4.1	0.34
	Cr24	4.6	3.5	1.14
Rayagada	Cr25	7.2	7.0	0.20
	Cr26	1.4	1.9	0.47
	Cr27	5.8	5.6	0.17
	Cr28	1.4	2.5	1.07
	Cr29	2.9	2	0.94
	Cr30	3	4.2	1.2
	Cr31	4.3	4.2	0.1
	Cr32	3.5	2.7	0.78
	Cr33	3.5	3.4	0.06
	Cr34	2	2.3	0.26
	Cr35	2	1.9	0.11
	Cr36	2.9	3	0.1
	Cr37	5.1	4.8	0.31
Angul	Cr38	4	3.9	0.14
	Cr40	2.8	3.1	0.32
	Cr41	3.5	3.6	0.08
	Cr42	4	3.3	0.67
	Cr43	2.7	2.2	0.48
	Cr44	1.4	2.1	0.72
	Cr45	1.5	1.4	0.08
	Cr46	3.6	4.2	0.58
	Cr47	3.4	3.9	0.53
Khurda	Cr48	3.5	3.7	0.24
	Cr49	2.9	3.7	0.83
	Cr50	6.6	6.2	0.35
	Cr51	5.2	5.4	0.17
	Cr52	2	1.8	0.16
	Cr53	5.4	4	1.41
	Cr54	1.6	0.7	0.88
	Cr55	4.3	4.2	0.08
	Cr56	3.9	4.2	0.3
	Cr57	4.9	4.4	0.53
Bhadrak	Cr59	6.2	6.2	0.03
	Cr60	3.8	4.3	0.52
	Cr61	5.1	4.2	0.87
	Cr63	5.6	5.3	0.31
	Cr64	6.1	6	0.09
	Cr65	4.5	4.5	0.01
	Cr67	3.2	2.3	0.91
	Cr68	4.3	4.5	0.24
	Cr69	3.5	3.7	0.21
Dhenkanal	Cr70	4.5	4.2	0.26
	Cr72	3.3	3	0.26
	Cr73	1.8	1.8	0.04
	Cr74	5.2	5.1	0.07
	Cr75	4.9	4.9	0.04
	Cr78	3.6	2.6	0.96
	Cr79	3.2	3.4	0.18
	Cr80	1.8	2.3	0.52
	Cr83	2.6	3.1	0.48
	Cr84	2.9	2.4	0.55
Cuttack	Cr86	3.8	3.8	0.01
	Cr87	1.7	1.9	0.23
	Cr88	3.5	2.7	0.81
	Cr89	2.9	2.8	0.11
	Cr90	2	2.5	0.53
	Cr91	0.9	2.4	1.46
	Cr92	5.9	6	0.05
	Cr93	1.5	1.2	0.3
	Cr94	5.9	5.9	0.04
	Cr95	3.3	3.3	0.05
	Cr96	1.6	1.1	0.52
Puri	Cr97	0.7	1.6	0.86
	Cr98	3.6	3.5	0.06
	Cr99	4	2.1	1.93
	Cr100	5.3	4.2	1.11
	Cr101	3.6	4.1	0.51
	Cr102	2.9	3	0.13
	Cr104	3.7	3.3	0.43
	Cr105	2.7	2.7	0.03
	Cr106	2.9	3.2	0.26
	Cr107	3.1	3.3	0.21
	Cr108	1.7	1.8	0.08
Jagatsinghpur	Cr109	2.3	2	0.29
	Cr110	1.2	1.9	0.7
	Cr111	2.3	2.1	0.2
	Cr112	1.5	1.6	0.05
	Cr114	3.3	3.6	0.26
	Cr115	2.4	2.5	0.13
	Cr116	4.6	5.3	0.72
	Cr117	3.2	2.9	0.27
	Cr118	3.8	3.2	0.6
	Cr119	2.1	2.5	0.39

Testing results of MLFN-8: In testing process, as shown in **Table 2** and **Figure 3B**, the comparison between predicted values and actual values are also very close, which means that the MLFN-8 model is precise while predicting.

**Table 2:** Predicted and experimental curcumin yield of test set data.

District	Accessionno.	Experimentalcurcumin yield(x_1_)	Predictedcurcumin yield(x_2_)	Absolute =Ix_1_-x_2_I
Kandhamal	Cr2	2	1.6	0.42
	Cr11	3.8	2.2	1.57
Gajapati	Cr15	1.6	1.3	0.33
Khurda	Cr58	5.6	4.3	1.26
Bhadrak	Cr62	5.9	4.3	1.64
	Cr66	1.8	4.2	2.38
Dhenkanal	Cr71	2.3	2.3	0.04
	Cr76	2.2	3.1	0.87
	Cr77	3.9	4.2	0.31
	Cr81	3	2.4	0.65
Cuttack	Cr85	3.4	2.4	1.04

Validation results of MLFN-8: the comparison result between predicted values and actual values is depicted in **Table 3**. A plot between target and output values is provided in **Figure 3C**. The relationship between predicted values and actual values implies that the validation process is precise.

**Table 3:** Predicted and experimental curcumin yield of validation set data.

District	Accessionno.	Experimentalcurcumin yield(x_1_)	Predictedcurcumin yield(x_2_)	Absolute =Ix_1_-x_2_I
Gajapati	Cr18	1.6	2	0.13
Angul	Cr39	3.1	2	1.3
Dhenkanal	Cr82	3.8	3	1.12
Puri	Cr103	3	2	1.04
Jagatsinghpur	Cr113	1.5	2	0.05

### Analysis of Parameters

The data obtained from soil analysis and climatic factors of 119 places were statistically analyzed using ANOVA test. ANOVA is a method of portioning variability into identifiable sources of variation and the associated degree of freedom in the model. Eleven control parameters were considered in the present study. Each factor affected the response to a varying degree.

#### Curcumin Content

Curcumin content from the rhizomes obtained from Rayagada was highest (7.2%) compared to the rhizome samples collected from other districts with a minimum from Kandhamal (0.4%). The content of curcumin in the dried rhizome of 60 turmeric accessions collected from different places covering 10 agroclimatic zones varied from 0.4 to 8.8% (Singh et al., 2013).

#### Effect of Altitude

All the 119 sites chosen for sampling of turmeric populations were at different geographical locations with altitude ranging from a minimum of 2.8 m (Puri) to a maximum of 872 m (Gajapati).

The curcumin content in the rhizomes sampled from 119 sites decreased progressively from low altitude to high altitude. The respective correlation coefficient (r) was -0.2 and reached statistical significance level (*P* < 0.20), which indicates that curcumin production is positively favored by low altitude.

#### Effect of Environmental Factors on Curcumin Content

The environmental factors recorded during the experiment showed a wide range of variation among different districts from where the samples were collected. At different districts the minimum average temperature ranged from 24.7°C to 17.5°C, maximum temperature ranged from 35.2°C to 29.2 °C, average rainfall varied from a minimum of 2.7 mm to a maximum of 5.2 mm. Similarly maximum and minimum relative humidity varied from 84.2 to 75.9% and 63.2 to 49.9% respectively. The variation in curcumin content was related positively with minimum relative humidity with *r* = 0.26 (*P*_min.rel.humidity_ < 0.44) whereas curcumin yield decreased with increase in maximum relative humidity with *r* = -0.15 (*P*_max.rel.humidity_ < 0.12). The correlation coefficient of curcumin content with average rainfall was -0.07 (significant at *P* < 0.55). The linear correlation coefficient (*r*) was 0.03 for maximum average temperature (significant at *P* < 0.71). The correlation coefficient of minimum average temperature was *r* = -0.03 (*P* < 0.54).

#### Effect of Soil pH

Soil pH of different districts mainly ranged between 6.05 (Bhadrak) to 7.97 (Angul). The correlation coefficient (*r*) between soil pH and curcumin content was -0.16 (*P* < 0.05). The results indicated that pH showed negative correlation with curcumin content of turmeric rhizome.

#### Effect of Soil Organic Carbon

Soil OC content of different districts mainly ranged between 3.27 kg/ha (Rayagada) and 0.42 kg/ha (Kandhamal). The correlation coefficient (*r*) between soil OC content and rhizome curcumin content was 0.04 (*P* < 0.08). The results indicated that OC showed positive correlation with curcumin content of turmeric rhizome.

#### Effect of Soil Nitrogen (N)

Soil nitrogen content of different districts mainly ranged between 623.7 kg/ha (Khurda) to 82.2 kg/ha (Jagatsinghpur). The correlation coefficient (*r*) between soil nitrogen content and curcumin yield was 0.18 and are statistically significant at *P* < 0.73 level. The results revealed that soil nitrogen positively favored curcumin content.

#### Effect of Phosphorous (P)

Among eight districts, total phosphorous content in the soil ranged from 374.2 kg/ha (Bhadrak) to 9 kg/ha (Rayagada). The correlation coefficient (*r*) between phosphorous and curcumin content was 0.08 and are significant at *P* < 0.42 level. The results demonstrated that soil phosphorous positively favored curcumin content in turmeric rhizomes.

#### Effect of Soil Potassium (K)

Total soil potassium content ranged between 1284 kg/ha to a maximum of 13.8 kg/ha in different districts. The correlation coefficient (*r*) between soil potassium and curcumin content was 0.03 and are significant at *P* < 0.27 level. This indicates that soil potassium also had a positive relationship with curcumin content.

From our result it is concluded that the variations in oil content were coupled with altitude, environmental variables and soil nutritional factors. This result is in close agreement with Sharma et al. (2000) and Alam and Naik (2009). Sharma et al. (2000) reported wide variation in climatic factors among the sites of collection of samples of *Podophyllum hexandrum* with variation in podophyllotoxin content. Alam and Naik (2009) developed ANN based prediction model in *Podophyllum hexandrum* for optimization of podophyllotoxin content. He showed that factors like soil pH, OC, nitrogen were more sensitive toward podophyllotoxin content. ANN model has also been developed in *Artemisia annua* for artemisinin content (Pilkington et al., 2014).

This is the first report demonstrating impact of soil nutrients and environmental factors on curcumin content of turmeric among 8 agro-climatic regions of Odisha using ANN model. Our work demonstrated that single factors are less effective on curcumin content than the combinations of factors. All the factors are correlated with the curcumin content, but single factors are not significantly effective as evidenced by their regression values showing little correlation with curcumin content. On the other hand, the ANN model developed combining all the factors showed highest correlation (*r* = 0.91) with curcumin content. Various research described that pH value influenced secondary metabolites production. Alkaloid production of *Lupunus polyphyllus* increased by cell culture when pH value decreased from 5.5 to 3.5 in the culture medium (Endress, 1994).

For metabolism of plants, soil organic matter provides N, P, K and essential metal cofactors. High soil organic matter content can uniformly supply the nutrition to plants, provide the plants a good growth and metabolic status and enhance the resistance of the plants to stresses (Alam and Naik, 2009). These are the basis of secondary metabolism. In our work all the soil factors altogether have significant effect on curcumin content.

### Response Surface and Contour Plots

**FIGURE 4:**
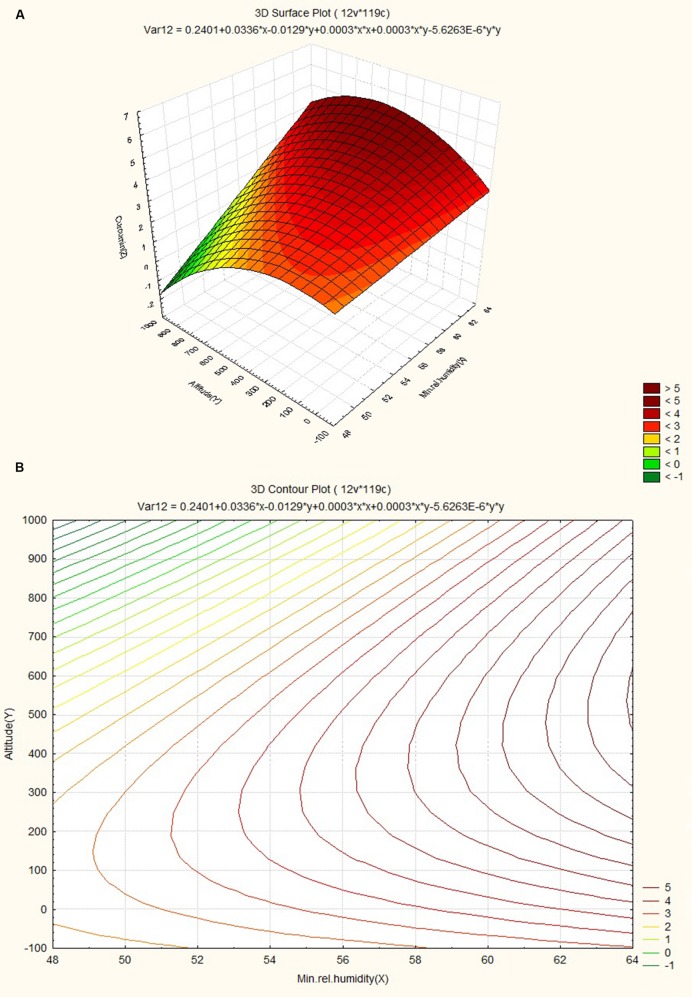
**(A)** Surface plot for combined effect of altitude and minimum relative humidity on curcumincontent. **(B)** Contour plot for combined effect of altitude and minimum relative humidity on curcumin content.

**FIGURE 5:**
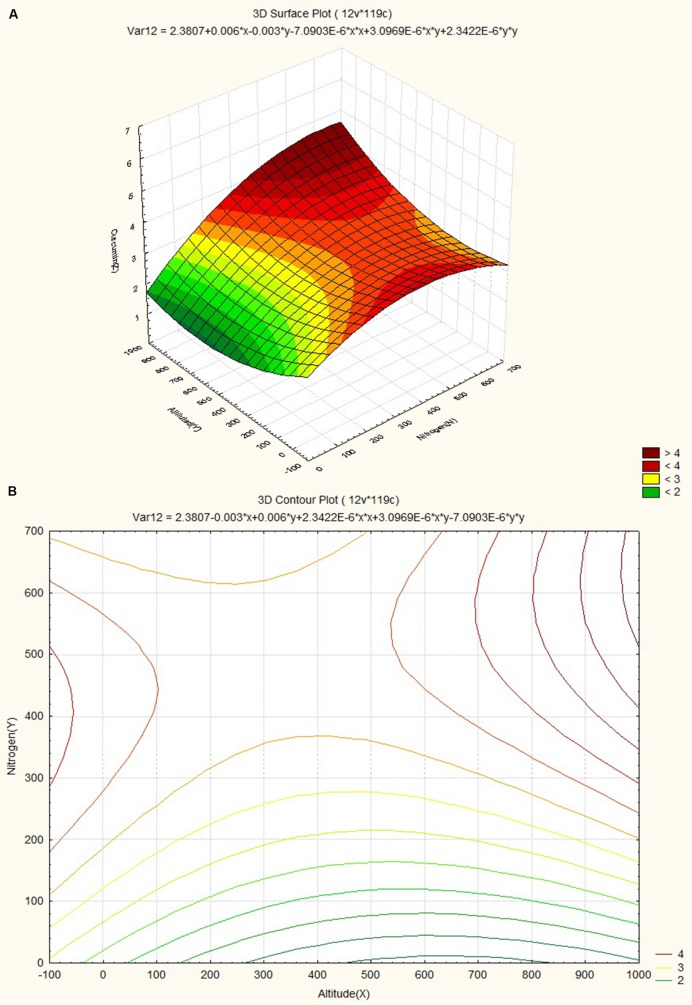
**(A)** Surface plot for combined effect of altitude and soil nitrogen content on curcumin content. **(B)** Contour plot for combined effect of altitude and soil nitrogen content on curcumin content.

**FIGURE 6:**
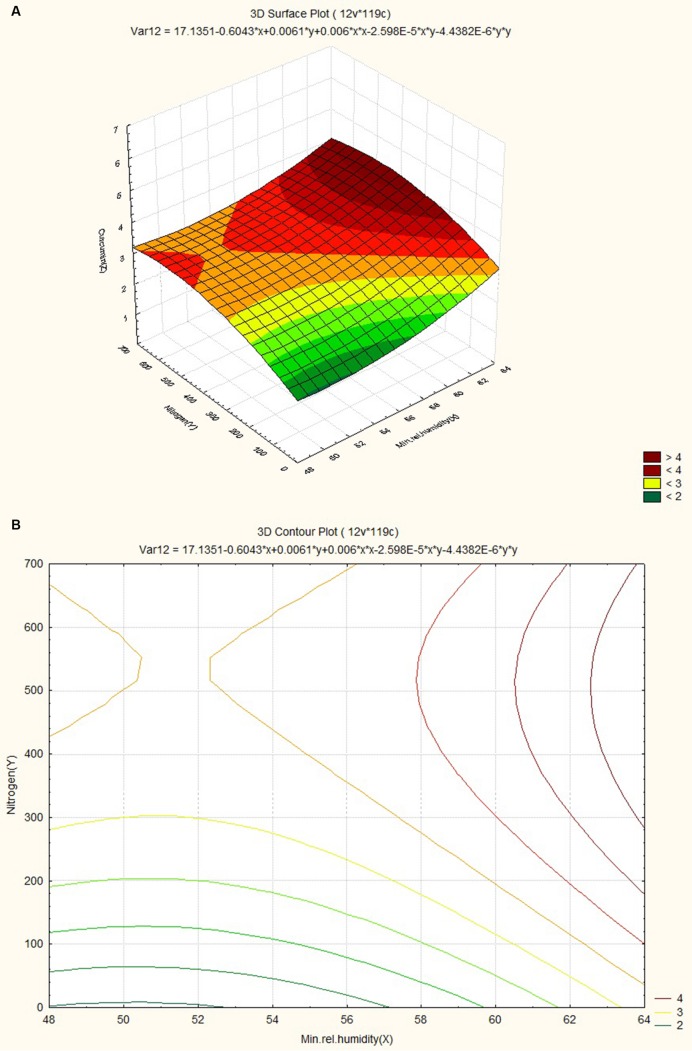
**(A)** Surface plots for combined effect of nitrogen and minimum relative humidity on curcumincontent. **(B)** Contour plot for combined effect of nitrogen and minimum relative humidity on curcumin content.

The response surface curves were plotted to understand the interaction of the variables and to determine the optimum level of each variable for maximum response. The response surface plot and contour plot for minimum relative humidity, altitude and curcumin content are shown in **Figures 4A,B** respectively. The figures show the combined effect of minimum relative humidity and altitude on curcumin content. The curcumin content increases with gradual increase in altitude value nearly up to 550 m and minimum relative humidity. The response surface plot and contour plot for altitude, nitrogen content of soil and curcumin content are shown in **Figures 5A,B** and they show the effect of altitude, and nitrogen content of the soil up to a certain range. The nitrogen content range lies between 200 and 600 kg ha^-1^ and the altitude range 0–100 m then from 540 m onward. The response surface plot and contour plot for minimum relative humidity, nitrogen content of soil and curcumin content are shown in **Figures 6A,B** and it shows the effect of minimum relative humidity and nitrogen content of soil on curcumin content. Curcumin content is increased with gradual increase in minimum relative humidity and nitrogen content of the soil.

**FIGURE 7:**
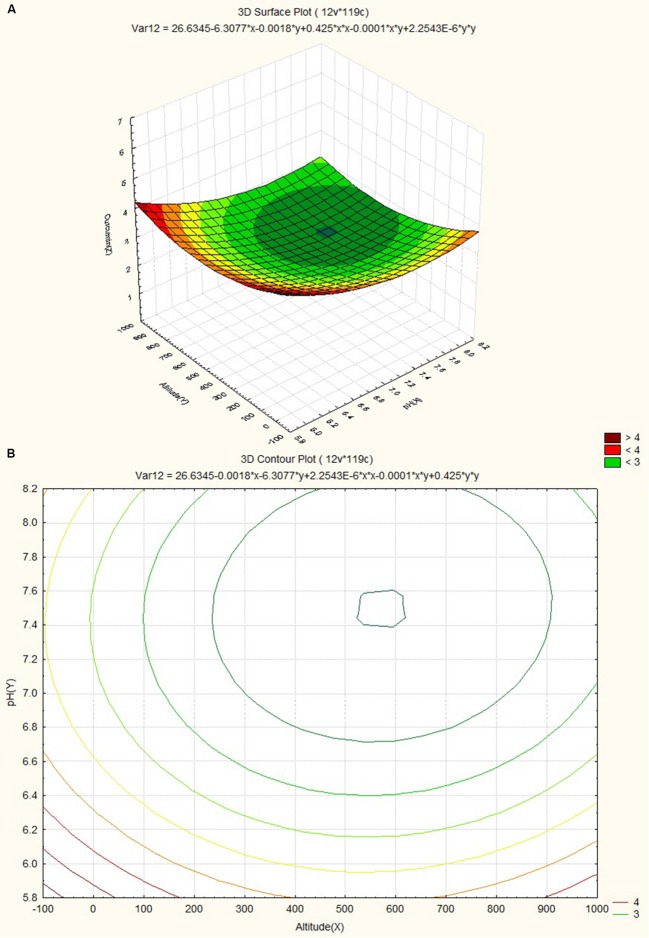
**(A)** Surface plots for combined effect of altitude and pH on curcumin content. **(B)** Contour plots for combined effect of altitude and pH on curcumin content.

The response surface plot and contour plot for soil pH, altitude and curcumin content are shown in **Figures 7A,B** and it shows the effect of pH of soil and altitude on curcumin content. Slightly acidic soil (pH value ~6.2) with low altitude favored curcumin content.

**FIGURE 8:**
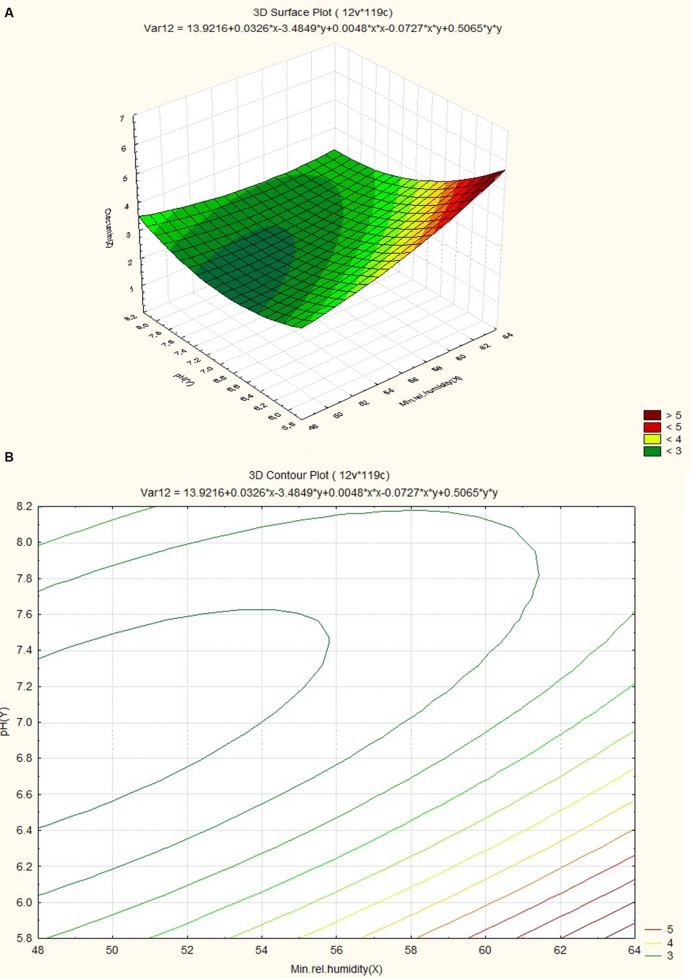
**(A)** Surface plot for combined effect of pH and minimum relative humidity on curcumin content. **(B)** Contour plot for combined effect of pH and minimum relative humidity on curcumin content.

**FIGURE 9:**
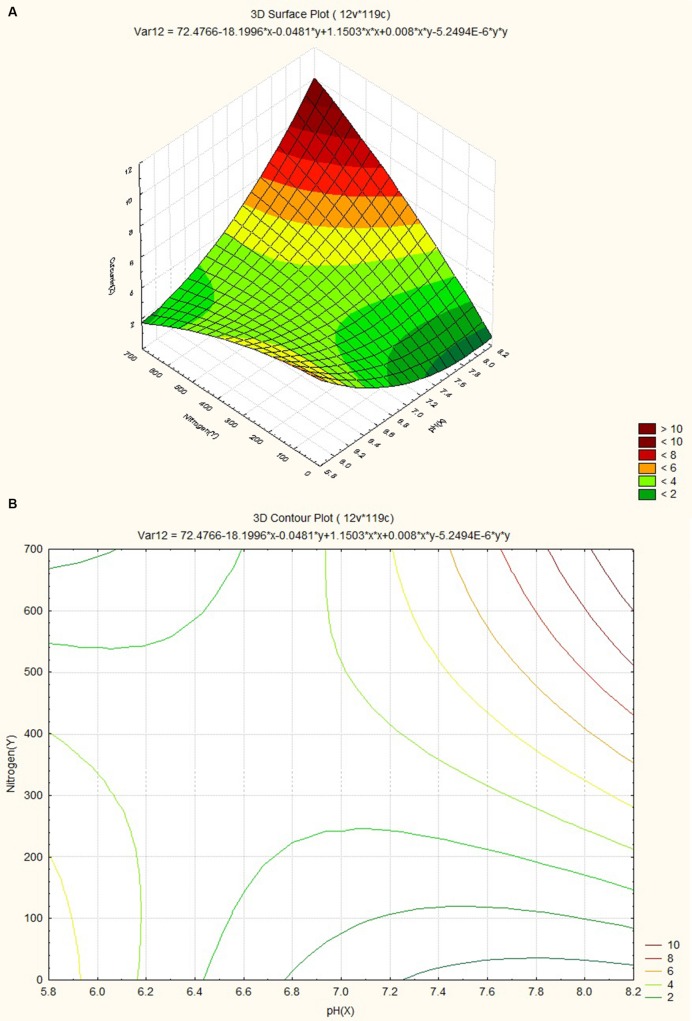
**(A)** Surface plot for combined effect of nitrogen and pH on curcumin content. **(B)** Contour plot for combined effect of nitrogen and pH on curcumin content.

The response surface plot and contour plot for minimum relative humidity, pH and curcumin content are shown in **Figures 8A,B** and it shows the effect of minimum relative humidity and pH on curcumin content. Curcumin content is favored by acidic pH (~6.2) and an increasing value of minimum relative humidity. The response surface plot and counter plot for pH, nitrogen content and curcumin content are shown in **Figures 9A,B** and they show the effect of pH and nitrogen content on curcumin content. The curcumin content is favored by the increase in both pH (in slightly alkaline range) and nitrogen content of the soil.

Several studies on the optimized conditions for the osmotic dehydration process using response surface plots have been published for papaya, potato, diced pepper and banana (El-Aouar et al., 2006; Ozdemir et al., 2008; Mercali et al., 2011; Maran et al., 2013).

### Sensitivity Analysis

**FIGURE 10:**
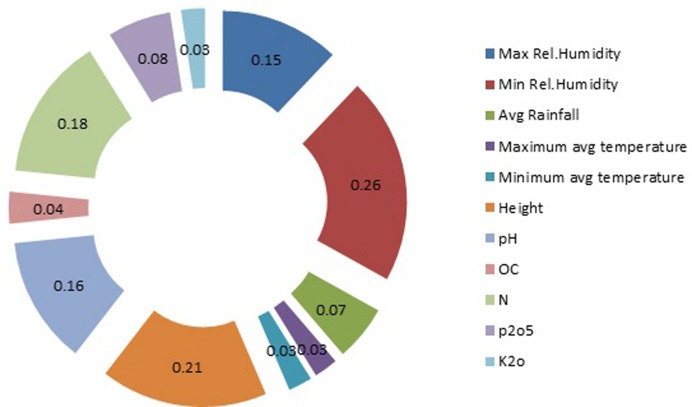
**Sensitivity analysis of input parameters on curcumin yield (output)**.

Results of the sensitivity analysis are shown in **Figure 10**. The calculated curcumin content showed the greatest sensitivity to minimum relative humidity with regression value of 0.26. The next greatest sensitivity was to altitude, followed by soil nitrogen content and pH with regression value of 0.21, 0.18, and 0.16 respectively. Maximum relative humidity came fifth in influencing the curcumin production. The calculated curcumin content showed considerably less sensitivity to the remaining 6 input factors (Relative Humidity, Avg Rainfall, Maximum avg temperature, OC, phosphorous content and Potasium content). A sensitivity analysis is the method of studying the behavior of a model, and assessing the significance of each input variable on the values of the output variable of the model. Sensitivity analysis provides insight into the usefulness of individual variables. By the help of this kind of analysis, it is possible to judge which inputs for curcumin content parameters should be considered as the most significant and least significant. To evaluate the predictive ability and validity of the developed models, a sensitivity analysis was performed with the best network for yield of curcumin. Dai et al. (2011) examined effect of soil moisture and salinity stress on sunflower yield by using ANN. Results of the sensitivity analysis indicated that at each level of salinity stress, at different soil moisture, the yield would vary. Pahlavan et al. (2012) carried out sensitivity analysis of input parameters on basil production. Sensitivity analysis revealed that chemical fertilizer; farm yard manure (FYM), diesel fuel and other chemical energies had the highest sensitivity on output; while the sensitivity of electricity, human labor and transportation energies was relatively low.

### Optimization of Curcumin Content

Curcumin content was analyzed in triplicate from 119 accessions of turmeric. It was found that the curcumin content from the rhizomes obtained from Rayagada was comparatively more (7.2%) than that in the rhizome samples collected from other districts with a minimum from 0.4%, (Kandhamal). The variation in curcumin content was significant among the districts. From the developed model it was found that the curcumin content in the rhizome sampled from these sites decreased progressively from low altitude. The respective correlation coefficient (*r*) was 0.04. The variation in curcumin content of turmeric is highly dependent on climatic factors. The variation in curcumin content was related positively with humidity; *r* = 0.02 (afternoon) and *r* = 0.08 (forenoon). The correlation coefficient between curcumin content was 0.01 with average rainfall. The linear correlation coefficient (*r*) was 0.002 for maximum temperature and 0.1 for minimum temperature and are positively correlated with curcumin content. The correlation coefficient (*r*) was 0.02 between soil OC and curcumin content. The results indicated that OC favored curcumin production in the turmeric rhizome.

**FIGURE 11:**
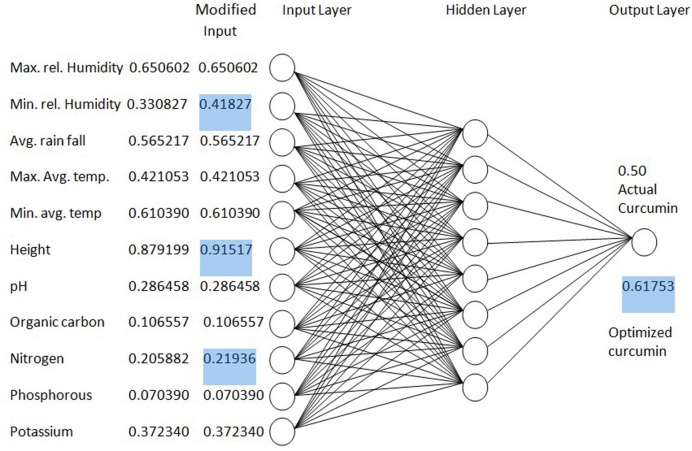
**Optimization of curcumin yield by changing parameters of ANN model**.

The result of the regression between soil pH and curcumin was 0.03. The correlation coefficients (*r*) was 0.05 between soil nitrogen content (N) and curcumin content. The statistical analysis results of the linear regression between phosphorous (P) and curcumin content was 0.06. The correlation content (*r*) was 0.003 between soil potassium (K) and curcumin content. According to the findings, curcumin content of turmeric can be improved through the soil management to create the soil conditions similar to the original ones. An optimization process using the earlier developed ANN model is shown in **Figure 11**.

Medicinal productivity of many medicinal herbs is more in their original habitat than in cultivated lands. Soil nutrient and environmental factors similar to original habitats must be most suitable for the active compound production. According to the results of this research, curcumin production of turmeric can be improved through the soil management to create the soil conditions similar to the original ones.

Further, plant secondary metabolism is a complex physiological process. The secondary metabolite production is influenced by the plant’s own physiological age, status and other environmental factors. So, the effects of soil on curcumin content of turmeric are more complicated. However, it is assumed that the variation in curcumin content is dependent on these factors, which needs further research.

### Prediction of Curcumin Content at a New Site

**FIGURE 12:**
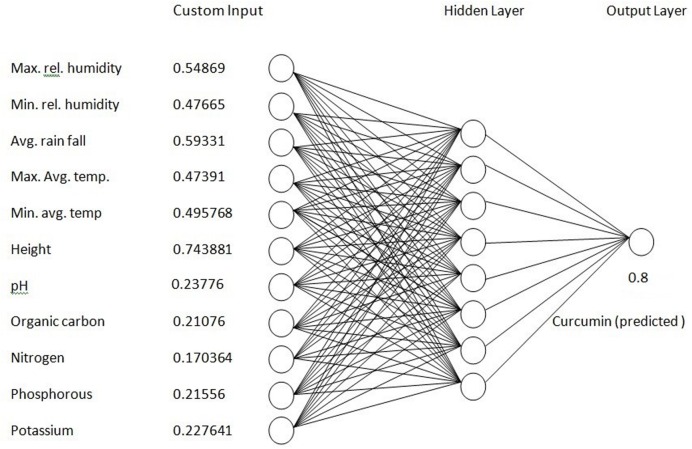
**Prediction of curcumin yield using ANN model**.

The prediction model (ANN) (**Figure 12**) developed in this study will be helpful to get maximum yield of curcumin in turmeric. This model could predict the curcumin content for a new site which is very close to the experimental value. The results demonstrated that using a combination of soil and environmental data, we were able to successfully predict curcumin content with the developed ANN model. The ANN model was 80% accurate when tested using the reserved data set. The same ANN model, without retraining was tested at Pottangi. This model predicted the curcumin content for Pottangi is 0.8 which is similar to the experimental value (**Figure 12**).

## Conclusion

The prediction model (ANN) developed in this study to map the effect of different environmental factors on curcumin content will be helpful to get maximum curcumin yield in tumeric. The results demonstrated that using a combination of soil and other environmental data, we were able to successfully predict curcumin content with ANN. Thus ANN model is very important for prediction and optimization of curcumin content of turmeric at a specific site for commercial cultivation.

## Author Contributions

Study conception and design: AA, AK, PN, and SN. Acquisition of data: AA, AK, IS, and SM. Analysis and interpretation of data: AA, AK, and AM. Drafting of manuscript: RJ. Critical revision: RJ, PN, and SN.

## Conflict of Interest Statement

The authors declare that the research was conducted in the absence of any commercial or financial relationships that could be construed as a potential conflict of interest.
